# Effects of prophylactic swallowing exercises on dysphagia and quality of life in patients with head and neck cancer receiving (chemo) radiotherapy: the Redyor study, a protocol for a randomized clinical trial

**DOI:** 10.1186/s13063-019-3587-x

**Published:** 2019-08-14

**Authors:** Anna Guillen-Sola, Neus Bofill Soler, Ester Marco, Oscar Pera-Cegarra, Palmira Foro

**Affiliations:** 1 Physical Medicine and Rehabilitation Department, Parc de Salut Mar (Hospital del Mar, Hospital de l’Esperança), Hospital de l’Esperança. Sant Josep de la Muntanya, 12, 08024 Barcelona, Catalunya Spain; 2 Rehabilitation Research Group, Institut Hospital del Mar d’Investigacions Mèdiques (IMIM), Barcelona, Catalunya Spain; 3 Physical Medicine and Rehabilitation Department, Hospital Verge de la Cinta, Tortosa, Catalunya Spain; 4 Radiotherapy Department, Hospital del Mar, Parc de Salut Mar, Barcelona, Catalunya Spain; 5 Radiation Oncology Research Group, GREOR. Institut Hospital del Mar d’Investigacions Mèdiques (IMIM), Barcelona, Catalunya Spain

**Keywords:** Deglutition, Deglutition disorders, Head and neck cancer patients, Rehabilitation, EAT 10

## Abstract

**Background:**

Radiation-induced dysphagia is common in patients with head and neck cancer (HNC). Available evidence suggests that exercise therapy prior to oncological treatment could potentially improve deglutition and quality of life; however, a randomized clinical trial is needed to confirm this observation.

**Methods/design:**

The Redyor study is a single-blind randomized clinical trial designed to compare the effect of prophylactic oropharyngeal exercises on quality of life and dysphagia of 52 patients with HNC referred to the Radiotherapy Department. The intervention will consist of respiratory muscle training (3 times/day, 5 days/week, 21 weeks) added to the standard swallow therapy. All patients will perform the same exercise intervention, but at different times: before chemoradiotherapy (CRT; early intervention group) or immediately after completing CRT (late intervention group). The main outcome will be change in dysphagia severity assessed with the Penetration-Aspiration Scale in videofluoroscopy study; quality of life will be assessed with the EORTC-QLQ-C30 and its Head and Neck Cancer Module (QLQ-H&N35) at 3, 6, and 12 months after completing CRT.

**Discussion:**

This ongoing clinical trial, registered in 2016, is based on the hypothesis that undergoing a pre-radiotherapy rehabilitation (pre-habilitation) program will have greater benefits (less decrease in quality of life, less delay in swallowing parameters, and less severe dysphagia) compared to post-CRT rehabilitation. The main objective is to assess dysphagia severity in HNC patients; and secondly, to evaluate changes in dysphagia-related quality of life, and to determine the correlation between a clinical variable and instrumental parameters during this period.

**Trial registration:**

NCT0209009911.

**Electronic supplementary material:**

The online version of this article (10.1186/s13063-019-3587-x) contains supplementary material, which is available to authorized users.

## Background

Squamous head and neck cancer (HNC) has high incidence in developed countries. HNC is categorized according to the area of the head or neck in which it occurs, with greater incidence in the larynx, followed by the oropharynx, oral cavity, and hypopharynx. At the time of diagnosis, 1% of all patients have a distant metastasis, with the highest rate (4%) observed in the nasopharynx and earlier-stage tumors located in the larynx and the oral cavity. Tobacco and alcohol use and human papilloma virus (HPV) infection are the most important risk factors [1].

Although chemo-radiation therapy (CRT) protocols have been designed to preserve swallowing function and essential speech organs, dysphagia is a frequent symptom in these patients and the primary adverse effects are usually associated with acute or late swallowing disturbances [2–6]. Preservation of underlying anatomical structures does not guarantee normal function. CRT affects target areas and may result in lack of coordination of swallowing phases, lack of swallow coordination with respiratory function, reduced elevation of larynx, delayed laryngeal closure, loss of tongue strength, and prolonged oral and pharyngeal time in swallowing [6–9]. Radiation-induced dysphagia pathogenesis includes an initial process of acute inflammation with the appearance of edema, which may be followed by fibrosis of the soft tissues resulting in neurological alteration and muscle damage. Xerostomia, pain, and pharynx obliteration are the key elements of acute-phase dysphagia. At 3 months, many patients have regained swallowing function. In later stages, with the appearance of diffuse fibrosis of the connective tissue and skin in the irradiated area, changes are observed in the efficacy and safety of swallowing. It is believed that hypoxia and chronic oxidative stress could perpetuate tissue damage even long after the end of treatment, which would explain the appearance of dysphagia in the chronic phase [10]. These side effects contribute to higher rates of malnourishment, weight loss, and bronchoaspiration [6, 11], resulting in a need for alternative or supplementary methods for feeding and hydration, both early and long-term [8].

For years, delays have been reported in referring HNC patients undergoing CRT to rehabilitation departments. Patients were only referred when they presented with obvious swallow deficits or the consequent malnourishment, weight loss, changes in voice characteristics, etc., often months or years post-CRT [12]. The greater the delay, the worse is the patient’s detrimental muscle disuse and swallow dysfunction [13, 14].

In recent years, there has been a growing interest in swallowing interventions. Potential benefits of prophylactic exercises conducted during [15–17], soon after [18], or before the CRT intervention [19] have been described, and improvements in functional swallow outcomes and quality of life parameters after respiratory therapy (RT) intervention have been reported [14]. Nevertheless, supportive care for earlier dysphagia management in rehabilitation departments continues to play a secondary role in HNC diagnosis in most health systems [9, 12].

New ways to treat HNC dysphagia in an early intervention are now being explored, using inspiratory and expiratory muscle training (IEMT), a technique initially developed for patients with chronic respiratory diseases [20] that has shown its usefulness in patients with dysphagia [21–25]. Designed to improve respiratory muscle strength, IEMT also trains muscles involved in coughing, speech, and swallowing [26]. The hypothesis of the ongoing Redyor (Rehabilitation Dysphagia Oropharyngeal Cancer) study is that improvement of the swallowing function is due to the strengthening of the suprahyoid muscle (anterior portion of the digastric, mylohyoid, and geniohyoid). The suprahyoid/submental muscles participate in the pharyngeal phase of swallowing. Their weakness or lack of coordination can decrease the amplitude of the hyoid, causing an inadequate opening of the upper esophageal sphincter that exposes the airway to the passage of the bolus. Some authors have evaluated the effect of IEMT on the swallow muscles by videofluoroscopy swallow studies (VFSS), noting that the amplitude of the hyoid movements increase during training, both in the oral (jaw and tongue) and pharyngeal phases [27]. The usefulness of IEMT to train respiratory, cough, speech, and swallowing muscles is well established in pathologies other than chronic obstructive pulmonary disease, such as stroke-related dysphagia [28], but the most recent neurophysiological findings suggest the capacity for improving motor recruitment of the suprahyoid musculature, the activity of pharyngeal musculature, and the palate, as well as an increase in the amplitude of the movements of the hyoid [29, 30], could be considered and included as a new therapeutic tool for the treatment of HNC swallowing disturbances in the acute stage after diagnosis.

Available data suggest that pre-habilitation exercises could further improve these results [18, 19]. The Redyor study is based on the hypothesis that an early intervention could contribute to preserve swallow function and quality of life. The main objective of this study is to determine the effectiveness of 21-week IEMT added to standard swallowing exercises performed before or immediately after CRT (early or late intervention, respectively) to preserve swallow function in patients with HNC receiving radiotherapy in a randomized clinical trial. Secondary objectives of this study include assessing changes in quality of life, comparing the effectiveness of the VFSS and Volume-Viscosity Swallow Test (V-VST) for screening dysphagia, and evaluating the effect of IEMT added to standard swallow exercises in a home-based dysphagia rehabilitation program in patients with HNC undergoing CRT.

## Methods/design

### Trial design

The Redyor study is a prospective randomized single-blind clinical trial aimed to determine the benefits of early rehabilitation to preserve swallow function and quality of life in patients with HNC receiving radiotherapy, following the Standard Protocol Items: Recommendations for Interventional Trials (SPIRIT) [31]. Throughout the recruitment period, patients who provide their informed consent (Additional file 1) to participate will be randomized to one of two groups: early intervention or post-CRT intervention. No major changes to methods after the start of the study are planned. This trial has been approved by the local Clinical Ethics Committee (Additional file 2).

### Settings and locations

**Fig. 1:**
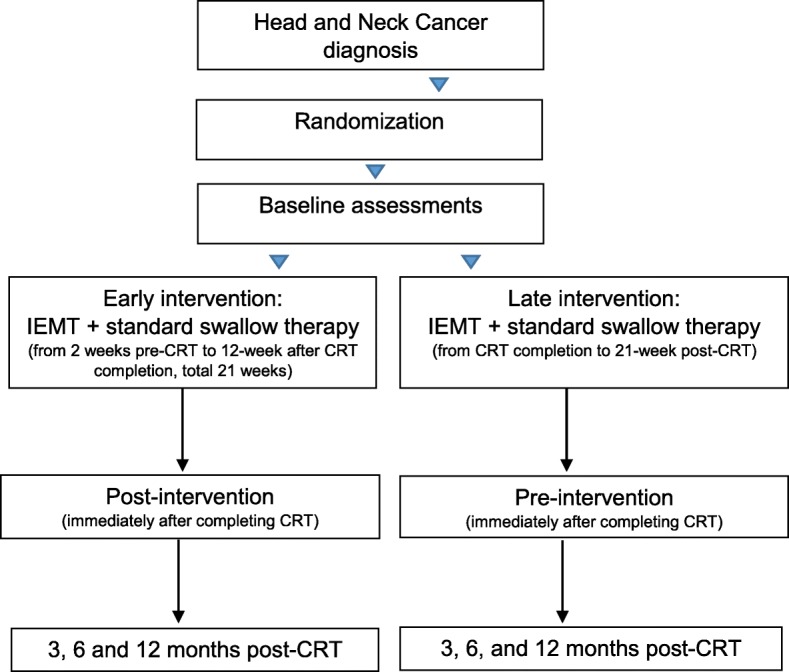
The Redyor study flow diagram

Recruitment will be carried out in the Radiotherapy Department at the Parc de Salut Mar (Hospital del Mar and Hospital de l’Esperança), Barcelona, Catalonia, Spain. Baseline dysphagia assessments will be performed in the Swallowing Disorders Unit of the Physical Medicine and Rehabilitation Department at Hospital de l’Esperança and all VFSS assessments will be conducted in collaboration with a radiologist. Statistical analysis will be done at the Hospital del Mar, in the Medical Research Institute (IMIM). Study sites and phases are summarized in the flow diagram (Fig. 1).

### Eligibility criteria

Patients with advanced HNC receiving radiotherapy will be eligible to participate in this clinical trial. Potential participants will be excluded if they have previous history of HNC and/or head or neck radiation therapy or surgical treatment, or of dysphagia due to causes other than cancer. Inclusion and exclusion criteria are listed in Table 1. During the study period, a patient will be excluded from the study if any of the following occurs: emergence of any of the exclusion criteria, onset of any disease or medical condition that will make it difficult for the patient to continue participation, decision to withdraw from the study for any reason, transfer out of the service area or death, impossibility to practice exercises due to medical condition.

**Table 1:** Inclusion and exclusion criteria of ReDyOr study

Inclusion Criteria	Exclusion criteria
Advanced HNC	Previous history of HNC
Receiving Radiotherapy	Previous head or neck radiation therapy
	Surgical treatment on HNC area
	dysphagia due to causes other than cancer

*HNC* Head and neck cancer

### Intervention

The training protocol consists of IEMT and standard swallowing exercises. Patients will be instructed to maintain a rate of 15–20 breaths/min using the Orygen-Dual® valve (described elsewhere in detail) [21, 22], a respiratory device that allows patients to train inspiratory and expiratory muscles simultaneously. Training loads will be set at a pressure equivalent to 10 maximal repetitions (if tolerated). These external pressures will be regulated weekly at 30% of maximal inspiration/expiration respiratory measures obtained. Patients will be instructed to perform 3 sets of 5 repetitions and standard swallowing exercises (mobility and tonicity exercises of the tongue, palate, larynx, and constrictor muscles), 3 times/day, 5 days/week, for 21 weeks. An experienced swallowing therapist will supervise IEMT weekly. The protocol will be the same for both groups, but one group will start 2 weeks before CRT and the other one will begin immediately after completing CRT (Fig. 1).

### Outcome measures

Main outcome variable:

- Change in dysphagia severity observed by VFSS and assessed with the Penetration-Aspiration Scale: scores 1–2 indicate normal swallowing, 3–5 penetration, and ≥ 6 aspiration

Secondary outcome variables:

- Change in quality of life, assessed with the European Organization of Research and Treatment of Cancer (EORTC) Quality-of-Life Questionnaire (QLQ-C30) and its Head and Neck Cancer Module (QLQ-H&N35).
- Mouth opening or the maximal interincisor opening (MIO) of the mouth, using the Therabite® range of motion scale.
- Maximum isometric tongue pressure in an anterior position.
- Maximum inspiratory and expiratory pressures (PImax and PEmax, respectively).
- Impaired security (tone of voice, coughing during or after eating, or desaturation > 3% compared to baseline pulse oximetry) and efficacy in oral and pharyngeal phases of swallowing with the use of the Volume-Viscosity Swallow Test.
- Subjective difficulty of swallowing assessed by a Visual Analogic Scale (VAS) ranging from 0 to 10; a low score indicates no difficulties in eating and a high score, no oral intake.
- Peak cough flow (PCF) will be used to evaluate the effect of IEMT on voluntary cough. It is measured with spirometer in liters/minute. Patients will be instructed to perform three repetitions of voluntary cough and the best one will be chosen.

### Participant timeline

**Fig. 2:**
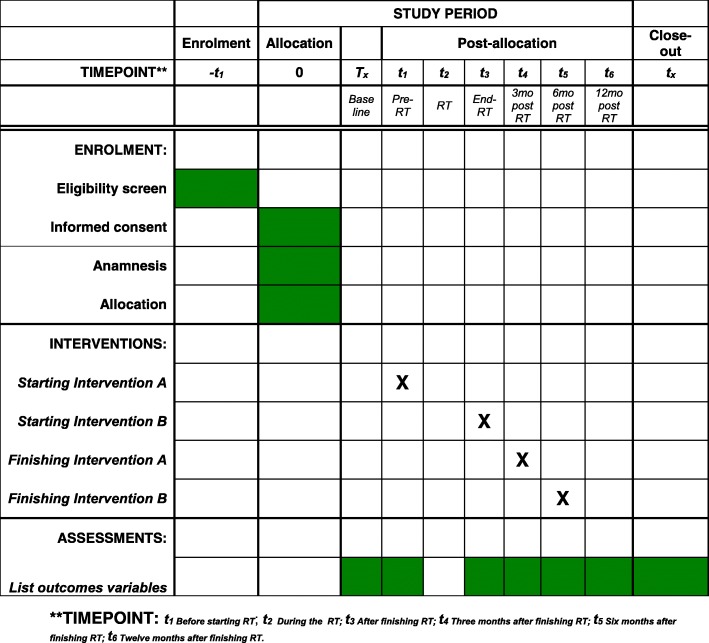
SPIRIT diagram of the project stages for the Redyor study

Eligible patients will be randomized and referred to the Rehabilitation Department for function assessment and intervention. The study outcomes will be assessed at baseline (2-week pre-CRT, t_0_), at the beginning of CRT (t_1_), at the end of CRT (t_3_), and thereafter at 3 months (t_4_), 6 months (t_5_), and 12 months (t_6_). The Redyor schedule of enrollment is shown in Fig. 2.

### Sample size calculation

Accepting an alpha risk of 0.05 and a beta risk of 0.2 in a two-sided test, a total sample size of 52 patients will be required to detect a difference ≥ 10 (SD 11) units in the test of quality of life and 2 (SD 2.3) units in the PAS. In order to account for estimated 20% loss to follow-up, five patients have been added, resulting 26 patients on each group.

### Assignment of interventions

#### Allocation

When a new patient meets the eligibility criteria, a researcher (PF) in the Radiotherapy Department will inform the researcher responsible for participant randomization (OPC), who will allocate the patient to one of the study groups using a simple random number generator program. The patient will then be referred to the Rehabilitation Department and the secretary responsible for scheduling the exercise intervention will be informed of the patient’s exercise timeline.

#### Blinding

The interdisciplinary researchers (EM, PF) will be blinded to study group assignments over the entire study period. After data analysis has been completed, results will be released to all patients and to participating clinicians and researchers.

### Data collection, management, and analysis

#### Data collection methods

The main outcomes will be collected by means of a Data Collection Logbook specially designed for this study. This logbook will include the EAT-10 and both quality of life questionnaires (EORTC-QLQ-C30 and QLQ-H&N35). One member of the investigation team (NBS) shall be responsible for the collection and processing of data related to main outcome variables. To promote participant retention, patients will perform a weekly supervised session; any patient not attending these scheduled sessions will be contacted by telephone.

#### Data management

An individual code will be used to identify each participant in this study. Only one of the two principal investigators (AGS) will have access to the database and shall be responsible for its management.

#### Statistical methods

Numerical variables will be expressed descriptively as mean and standard deviation (SD). Student *t*-test for paired data will be used to compare the quantitative variables before and after the intervention, and Student *t*-test for independent samples for inter-group comparison of pre- and post-intervention changes. At a later stage, mixed models will be performed to test changes in parameters and the between-group differences in evolution, adjusting by possible confounders. The evaluation of the correlations between variables will be based on the quantitative variables obtained from the various studies and bivariate (ordered Spearman correlation) and multivariate (multiple regression) analysis. Reasons for protocol non-adherence will be registered. The level of alpha risk accepted for all tests will be 0.05.

The evaluation of the correlations between variables will be based on the quantitative variables obtained from the various studies and bivariate (ordered Spearman correlation) and multivariate (multiple regression) analysis. Reasons for protocol non-adherence will be registered and missing data will be discarded. The level of alpha risk accepted for all tests will be 0.05.

#### Monitoring

Given the non-pharmacological nature of this study, a data monitoring committee is not needed. Any adverse effect related to the exercise intervention or any unintended effect will be registered.

### Ethics and dissemination

National and international research ethics guidelines will be followed, including the Deontological Code of Ethics, Declaration of Helsinki, and current confidentiality laws concerning personal data in Spain (*Ley Orgánica 3/2018*, 5 December) and the European Union (*European Parliament and Council Regulation EU 2016/619*). Detailed, understandable oral and written information will be provided to patients and family members, and informed consent to participate will be signed by all participants (Additional file 1). The Redyor study protocol and the informed consent process have been reviewed and approved by the Clinical Ethics Committee of the Institut Hospital del Mar d’Investigacions Mèdiques, Barcelona, Spain (Comité Ètic d’Investigació Clínica Parc de Salut Mar: reference number 2015/6288/I) (Additional file 2). Standard Protocol Items: Recommendations for Interventional Trials (SPIRIT) guidelines will be followed throughout [32] (Additional file 3). This trial is supported by the Asociación Española contra el Cáncer (AECC) and was registered at www.clinicaltrials.gov with code NCT0209009911 on February 9, 2016. Amendments to the original submitted protocol are subject to further ethics review and will be communicated to investigators and participants upon approval. Investigators and sponsor will communicate trial results to participants and healthcare professionals. Manuscripts will be prepared for submission to medical journals related to this field in order to contribute to HNC knowledge.

## Discussion

Rehabilitation intervention in acute HNC patients is currently limited in Spain. Swallow disturbances are considered a side-effect during CRT, and early swallowing and speech intervention is not systematically considered. Acute side-effects are considered “*normal*” in the development of the illness and during treatment, and the Rehabilitation Department becomes involved only when a patient demands a referral or a clinician requests an evaluation; decision protocols have not yet been developed. On the other hand, there is increased interest in facing this problem, first by developing a good screening method to determine which patients require evaluation, and then evaluating new treatment techniques, including home-based training interventions. A challenge when dealing with dysphagia related to HNC is determining the clinical profile of patients who might benefit from rehabilitation interventions (e.g., IEMT). Research is urgently needed to identify the usefulness of various dysphagia screening methods and therapeutic interventions such as IEMT.

### Strengths and limitations of the study

The study has several potential limitations that must be considered. First, losses to follow-up are common in cancer studies; to address this concern, sample size estimation assumed a loss of about 20% of patients (higher than the usual 10–15% used in previous studies). We presume that these losses will affect both study arms equally. Second, the study lacks a control group; however, this design is justified by the available evidence of the benefits of swallow and speech therapy in patients with HNC-related dysphagia.

### Repercussions of the Redyor study

Interventions to help patients confront, manage, and treat dysphagia are urgently needed. The lack of randomized controlled trials in the early diagnosis of HNC and the high number of patients lost to follow-up due to CRT side effects highlight the potential scientific contributions of this study.

## Trial status

Protocol version number: Redyor_2

Begin recruitment: 03/25/2015

End recruitment: 08/21/2018

End data collection: 04/29/2019

Trial status: Enrollment is in progress; final data collection will end May 2019

## Additional files

Additional file 1: Informed Consent for Redyor Study. (PDF 90 kb)

Additional file 2: Clinical Ethics Committee of the Institut Hospital del Mar d’Investigacions Mèdiques. (PDF 446 kb)

Additional file 3: SPIRIT 2013 Checklist: Recommended items to address in a clinical trial protocol and related documents. (DOC 122 kb)

## Data Availability

The datasets generated and/or analyzed during the current study are not publicly available due to the amount of data generated but are available from the corresponding author on reasonable request.

## Abbreviations

CRT: Chemo-radiotherapy treatment
VFSS: Videofluoroscopy swallowing study
