# Supplementary material for: Effects of prophylactic swallowing exercises on dysphagia and quality of life in patients with head and neck cancer receiving (chemo) radiotherapy: the Redyor study, a protocol for a randomized clinical trial
Source: Trials. 2019 Aug 14;20:503. doi: 10.1186/s13063-019-3587-x (PMC6694466; doi:10.1186/s13063-019-3587-x)
Supplement: Supplementary file 1 — Informed Consent for Redyor Study. (PDF 90 kb) [file 13063_2019_3587_MOESM1_ESM.pdf]

HOJA DE INFORMACIÓN Y CONSENTIMIENTO INFORMADO

Los médicos del Servicio de Radioterapia y Rehabilitación están realizando un estudio de investigación sobre beneficios de la rehabilitación profiláctica de la musculatura deglutoria en la calidad de vida y la deglución en los pacientes sometidos a quimio-radioterapia (QT/RT) por cáncer de cabeza y cuello. Entre los objetivos de este estudio, se encuentra determinar el impacto en la calidad de vida y la alteración de la deglución observada en dos programas rehabilitadores, así como comprobar la sostenibilidad del efecto beneficioso.

Usted ha sido seleccionado para participar en este estudio por haber sido diagnosticado de un cáncer de cabeza y cuello. El objetivo del estudio es evaluar el beneficio que puede aportar el entrenamiento de músculos deglutorios en la evolución de la enfermedad y en la minimización de las secuelas.

El propósito de esta información es solicitarle la participación en este estudio, ofreciendo la información necesaria para que usted pueda tomar la decisión, libre y voluntaria, de autorizar o rechazar la participación como voluntario en este estudio. En este sentido le comunicamos que informarle y solicitar su autorización es una norma de obligado cumplimiento para el/la médico que le atiende.

Si acepta a participar en este estudio supondrá un doble beneficio, tanto para Usted como para otros enfermos; el estudio nos permitirá conocer mejor cual es el tipo de tratamiento más adecuado para cada paciente, sin poder asegurar una mejora absoluta tras la aplicación del tratamiento.

El objetivo de este documento es que Usted conozca los detalles de las maniobras diagnósticas y terapéuticas que se le practicarán, antes de decidir su participación en este estudio. Este proceso se denomina consentimiento informado.

RESUMEN DEL ESTUDIO

La literatura describe ampliamente el deterioro en la calidad de vida del paciente sometido a QT/RT por un proceso oncológico de la zona de cabeza y cuello, produciéndose un pico de máxima intensidad durante los días inmediatamente posteriores al tratamiento, y presentando una mejora de forma gradual, sin conocerse su alcance ni límite temporal. Estudios recientes sugieren que la realización de rehabilitación previa al tratamiento oncológico podría mejorar potencialmente la calidad de vida deglutoria.

Este estudio pretende determinar el impacto en la calidad de vida y la alteración de la deglución observada en los dos programas rehabilitadores, así como el efecto en el tiempo. Así mismo, se evaluaran sistemas de cribaje clínicos para ayudar en la detección precoz del deterioro de la calidad de vida.

Además de las pruebas convencionales que se realizan para su diagnóstico y posterior tratamiento, se realizará una evaluación clínica de la deglución y musculatura deglutoria para detectar posibles trastornos de la deglución previos y que fases de la deglución quedan más afectadas durante el proceso de tratamiento activo.

El entrenamiento que usted realizará consiste en ejercicios para reforzar la musculatura oral, el tragar y la fuerza de la lengua, añadiéndose técnicas de incremento sensorial una vez finalizada la RT/QT. Todo ello se realizará en el Servicio de Rehabilitación, en una primera sesión de instrucción individual de 60 min y posteriormente 1 sesión semanal de 30 minutos para reforzar la realización de los ejercicios en domicilio. Los ejercicios instruidos deberá realizarlos en casa cada día (15/20 min).

ALEATORIZACIÓN
Todos los pacientes recibirán entrenamiento deglutorio, pero puede variar su intensidad. Un grupo empezará antes del inicio de la RT y otro al finalizar ésta, siendo común una parte del tratamiento. La duración será de 3 meses y la asignación a un grupo u otro de tratamiento se realizará por sorteo.

PRIVACIDAD
Durante el estudio se seguirán las directrices nacionales e internacionales (Código Deontológico, Declaración de Helsinki) para la investigación en seres humanos. Todo el personal que participa en el estudio respetará su intimidad. Su historia clínica es confidencial. Su nombre y demás datos personales no serán proporcionados a personas no autorizadas (Ley Orgánica 15/1999 de 13 de diciembre) de Protección de Datos de Carácter Personal (LOPD).

DERECHO DE INTERRUMPIR EL ESTUDIO
La decisión de participar en el estudio es del paciente. Los médicos salvaguardarán en todo momento su bienestar y velarán para que tenga el mínimo de molestias. Si tiene alguna duda debe preguntar a su médico. Si decide interrumpir el estudio en algún momento, se halla en plena libertad de hacerlo.

Los profesionales responsables del estudio son:

- Hospital de l'Esperança: Dra Palmira Foro, Dra. Anna Guillén-Solà, Dra Neus Bofill

Cualquiera de ellas estará capacitado para responder las preguntas que Usted tenga.

Yo (nombre y apellidos) ____ declaro que:

- He leído la hoja de información del estudio que se me ha entregado
- He podido hacer preguntas sobre el estudio
- He recibido suficiente información sobre el estudio
- He estado informado por (nombre del investigador) ___________________________

Comprendo que mi participación es voluntaria y que puedo retirarme del estudio:
- Cuando quiera
- Sin dar explicaciones
- Sin que ello repercuta en la atención médica recibida

Declaro haber sido informado que mis datos serán tratados de conformidad con lo que establece la L.O. de 13 de diciembre y de Protección de Datos de Carácter Personal (artículo 3, punto 6 del RD 223/2004).

Consiento que los datos clínicos referentes a mi enfermedad sean almacenados en un fichero automatizado que podrá ser manejado exclusivamente con fines científicos.

Por tanto: **doy mi consentimiento** a participar en el estudio que se me propone

Firma del médico responsable

Dr/a.:
Núm. Col.:
Barcelona, a __ de __________del 201_

Firma del voluntario

Sr/a.:

He decidido **no autorizar** a participar en el estudio que se me ha propuesto

Firma del médico responsable

Dr/a.:
Núm. Col.:
Barcelona, a __ de _________ del 201_

Firma del voluntario

Sr/a.:
