# Supplementary material for: Effects of prophylactic swallowing exercises on dysphagia and quality of life in patients with head and neck cancer receiving (chemo) radiotherapy: the Redyor study, a protocol for a randomized clinical trial
Source: Trials. 2019 Aug 14;20:503. doi: 10.1186/s13063-019-3587-x (PMC6694466; doi:10.1186/s13063-019-3587-x)
Supplement: Supplementary file 2 — Clinical Ethics Committee of the Institut Hospital del Mar d’Investigacions Mèdiques. (PDF 446 kb) [file 13063_2019_3587_MOESM2_ESM.pdf]

Additional file 2: Redyor study protocol approved by Clinical Ethics Committee of the Institut Hospital del Mar d’Investigacions Mèdiques

**CONFIRMATION**

**I confirm that the "Parc de Salut MAR - Ethics Committee" has reviewed and approved**

- ✓ The clinical study entitled: *"Valoración de los beneficios de la rehabilitación profiláctica de la musculatura deglutoria sobre la calidad de vida y deglución, a corto y medio plazo, de los pacientes sometidos a radioterapia por cáncer de cabeza y cuello: Ensayo Clínico Aleatorizado"*.

In which Dr. Palmira Foro Arnalot is the principal investigator.

- ✓ and accompanied patient information sheet and informed consent form

under the reference number: 2014/5707/I

at the meeting of: December 22nd, 2014

COMITÈ ÈTIC D'INVESTIGACIÓ CLÍNICA
CEIC - PARC DE SALUT MAR

---------------------------------
María Teresa Navarra Alcrudo
Secretary
Ethics Committee – Parc de Salut MAR

Barcelona, January 22nd, 2015

CEIm – Parc de Salut MAR
Dr. Aiguader, 88 I 08003 Barcelona I Telèfon 93 316 06 77 I Fax 93 316 06 36
ceic-psmar@imim.es | www.parcdesalutmar.cat
